# Reliability and Validity of the Japanese Version of the Kinesthetic and Visual Imagery Questionnaire (KVIQ)

**DOI:** 10.3390/brainsci8050079

**Published:** 2018-05-02

**Authors:** Hideki Nakano, Takayuki Kodama, Kazumasa Ukai, Satoru Kawahara, Shiori Horikawa, Shin Murata

**Affiliations:** Department of Physical Therapy, Faculty of Health Science, Kyoto Tachibana University, 34 Yamada-cho, Oyake, Yamashina-ku, Kyoto-city, Kyoto 607-8175, Japan; kodama-t@tachibana-u.ac.jp (T.K.); a901014011@tachibana-u.ac.jp (K.U.); a901014017@tachibana-u.ac.jp (S.K.); a901014050@tachibana-u.ac.jp (S.H.); murata-s@tachibana-u.ac.jp (S.M.);

**Keywords:** motor imagery, kinesthetic and visual imagery questionnaire, Japanese

## Abstract

In this study, we aimed to (1) translate the English version of the Kinesthetic and Visual Imagery Questionnaire (KVIQ), which assesses motor imagery ability, into Japanese, and (2) investigate the reliability and validity of the Japanese KVIQ. We enrolled 28 healthy adults in this study. We used Cronbach’s alpha coefficients to assess reliability reflected by the internal consistency. Additionally, we assessed validity reflected by the criterion-related validity between the Japanese KVIQ and the Japanese version of the Movement Imagery Questionnaire-Revised (MIQ-R) with Spearman’s rank correlation coefficients. The Cronbach’s alpha coefficients for the KVIQ-20 were 0.88 (Visual) and 0.91 (Kinesthetic), which indicates high reliability. There was a significant positive correlation between the Japanese KVIQ-20 (Total) and the Japanese MIQ-R (Total) (r = 0.86, *p* < 0.01). Our results suggest that the Japanese KVIQ is an assessment that is a reliable and valid index of motor imagery ability.

## 1. Introduction

Motor imagery is commonly defined as the mental simulation of one’s own performance without any associated overt movement [1]. Previous studies have suggested that motor imagery increases motor skill acquisition [2] and muscle strength [3] in healthy participants. Moreover, it is indicated that motor imagery is an effective rehabilitation tool for patients with various diseases involving the central nervous system or acute injuries requiring orthopedic interventions, including subacute stroke [4,5,6,7,8], chronic stroke [9,10,11], traumatic brain injury [12], multiple sclerosis [12], shoulder impingement syndrome [13], postsurgical injury of the anterior cruciate ligament [14], postsurgical flexor tendon repair [15], burn injury [16], phantom limb pain [17], complex regional pain syndrome [17,18], and motor coordination problems [19]. Regarding the neural mechanisms, motor imagery appears to activate the fronto-parietal network, as well as subcortical and cerebellar regions, which are also associated with actual motor execution [20]. 

Typically, motor imagery is subdivided into two different modalities: visual imagery and kinesthetic imagery [21,22]. These modalities have been defined previously by Guillot et al. [21]. Visual imagery requires self-visualization of a movement from a first-person (internal visual imagery) or third-person (external visual imagery) perspective. The first-person perspective corresponds to the representation of a movement as if the individual is conducting the action themselves, suggesting that they would visualize the movement as if they had a camera on their head. In contrast, the third-person perspective corresponds to the representation of movements as if the participant was a spectator and someone (either the individual or another person) performed the action. Kinesthetic imagery requires one to “feel the movement”, and to mentally perceive muscle contractions and stretching.

Previous research suggests that healthy subjects can be classified into visual-dominant and kinesthetic-dominant groups [23]. Moreover, motor learning performance appears to be more efficient for visual-dominant individuals when they are instructed to direct their attention externally. In contrast, motor learning performance is more efficient for kinesthetic-dominant individuals when they were instructed to direct their attention internally [23]. Furthermore, a similar phenomenon has also been observed in stroke patients [24]. Such results suggest it is important to evaluate motor imagery ability in individuals to determine the appropriate motor learning strategy.

The Kinesthetic and Visual Imagery Questionnaire (KVIQ) [25] is a representative tool to assess motor imagery ability. The KVIQ can be used to assess healthy individuals, as well as those with physical disabilities. It allows easy evaluation of motor imagery ability in a sitting position with single joint motions. Furthermore, the KVIQ assesses both visual and kinesthetic dimensions of motor imagery. The KVIQ is not self-administered, rather it is administered by a trained assessor. It assesses the vividness of each dimension of motor imagery (clarity of the image/intensity of sensation) on a 5-point ordinal scale. There are multiple versions of the KVIQ, including the KVIQ-20, which consists of 20 items (10 items each for visual and kinesthetic subscales), and the short version, KVIQ-10, which is a subset of the KVIQ-20, consisting of 10 items (5 items each for visual and kinesthetic subscales). The KVIQ-20 total score ranges from 20 to 100 (visual and kinesthetic subscale scores each range from 10 to 50). Meanwhile, the KVIQ-10 total score ranges from 10 to 50 (visual and kinesthetic subscale scores each range from 5 to 25). Higher scores indicate greater aptitude in motor imagery. Globally, both the KVIQ-20 and KVIQ-10 are widely used to evaluate motor imagery ability [26,27,28], and they have already been translated into German [29]. However, the KVIQ has not yet been adopted for Japanese speakers. Developing a Japanese version of the KVIQ is important to promote motor learning strategies focused on motor imagery, and to contribute to treatment for Japanese individuals with physical disabilities.

The Movement Imagery Questionnaire-Revised (MIQ-R) [30] is a questionnaire to assess motor imagery ability that was developed as a short version of the Movement Imagery Questionnaire (MIQ) [31]. The MIQ consists of 18 items (9 items each for visual and kinesthetic subscales), and the short version, the MIQ-R, consists of 8 items (4 items each for visual and kinesthetic subscales). The MIQ-R total score ranges from 8 to 56 (visual and kinesthetic subscales score each range from 4 to 28).

The two main differences between the KVIQ and MIQ/MIQ-R are the mode of administration and the selection of movements to be imagined [29]. Whereas the MIQ/MIQ-R is self-administered and focuses on complex high-level body movements, the KVIQ requires an examiner to be present to give instructions, perform example movements, and complete the scoring sheet. Moreover, the KVIQ focuses on simple, one joint axis movements of the upper and lower limbs, head, and trunk in a sitting position.

In the present work, we aimed to translate the English version of the KVIQ into Japanese, and to investigate the reliability and validity of the new Japanese version of the KVIQ.

## 2. Materials and Methods

### 2.1. Ethics Statement

This study was conducted according to the principles of the Declaration of Helsinki and was approved by the local institutional ethics committee (Kyoto Tachibana University) (approval number 17–13). All participants provided informed written consent and were free to withdraw from the study at any time.

### 2.2. Translation Procedure

Before translating the KVIQ, we first obtained authorization for the translation from the original author. The translation was conducted using a forward-backward method [32]. Initially, the KVIQ was translated from English into Japanese. Next, the forward-translation version was back-translated from Japanese to English. Subsequently, the back-translation version was checked and a preliminary Japanese version of the KVIQ was created. Finally, the preliminary Japanese version of the KVIQ was administered to three native Japanese participants. Based on the feedback gained from these participants, a final Japanese version of the KVIQ was created.

### 2.3. Participants

For this study, we enrolled 28 healthy participants (13 female and 15 male; mean age ± standard deviation: 20.64 ± 0.62 years). All participants were university students and were right-handed according to the Edinburgh Handedness Inventory (laterality quotient = 100) [33]. Participants were excluded if they had any chronic orthopedic, neurological, or psychiatric disease that might influence the results.

### 2.4. Measurement Procedure

To investigate the criterion-related validity of the Japanese KVIQ, we assessed participants’ motor imagery abilities with the Japanese KVIQ and the Japanese MIQ-R. The MIQ-R was selected as the gold-standard test because there is high validity between the KVIQ and MIQ-R [34]. Hereafter, the KVIQ and MIQ are referred to as the Japanese version of the task. Participants were tested in a quiet room with the door closed.

Administration of the KVIQ followed procedures outlined in previous studies [25,35,36]. First, the participant was asked to assume the starting position. The movement was then described and the participant was asked to perform it. Subsequently, the participant returned to the starting position and imagined the movement that they had just executed (the examiner verified that there was no actual movement). Finally, the participant was asked to rate the ease/difficulty with which they imagined the movement on the 5-point ordinal scale, and the clarity of the visual image or the intensity of the sensations associated with the imagined movement. 

The MIQ-R was measured with the Japanese version created by Hasegawa et al. [37] and the assessment was conducted according to previous studies [30,36,37]. Participants were asked to read the instructions carefully and ask the examiner any questions if necessary. Participants first performed the movement physically, and then completed visual or kinesthetic imagery of the movement. Finally, they recorded the score themselves on the score sheet. The MIQ-R consists of 8 items (4 visual and 4 kinesthetic), and each item was scored on a 7-point ordinal scale. 

### 2.5. Statistical Analysis

We calculated the Cronbach’s alpha value to investigate the internal consistency of the KVIQ-20/KVIQ-10. Moreover, we analyzed the relationship between the KVIQ-20/KVIQ-10 and the MIQ-R with Spearman’s rank correlation coefficients to investigate their criterion-related validity. All statistical analyses were performed in SPSS ver. 24.0 (IBM, Chicago, IL, USA), with the level of significance set at α = 0.05.

## 3. Results

Table 1 indicates the scores obtained for the KVIQ-20/KVIQ-10 and the MIQ-R. Cronbach’s alpha values for the KVIQ-20 were 0.88 (Visual) and 0.91 (Kinesthetic), and for the KVIQ-10 were 0.78 (Visual) and 0.77 (Kinesthetic) (Table 2). Furthermore, Table 3 indicates the results of the correlation analysis between the KVIQ-20/KVIQ-10 and the MIQ-R. We observed significant positive correlations between the KVIQ-20 (Visual) and the MIQ-R (Visual), between the KVIQ-20 (Kinesthetic) and the MIQ-R (Kinesthetic), and between the KVIQ-20 (Total) and the MIQ-R (Total) (r = 0.64, *p* < 0.01; r = 0.77, *p* < 0.01; and r = 0.86, *p* < 0.01, respectively). Moreover, we observed significant positive correlations between the KVIQ-10 (Visual) and the MIQ-R (Visual), between the KVIQ-10 (Kinesthetic) and the MIQ-R (Kinesthetic), and between the KVIQ-10 (Total) and the MIQ-R (Total) (r = 0.62, *p* < 0.01; r = 0.78, *p* < 0.01; and r = 0.90, *p* < 0.01, respectively).

## 4. Discussion

In this study, we investigated the reliability and validity of a Japanese version of the KVIQ. Our results suggest that the KVIQ has high internal consistency. Moreover, a significant positive correlation was observed between the KVIQ and the MIQ-R. Therefore, this study supports the KVIQ as a reliable and valid tool to assess motor imagery ability.

The scores of the KVIQ-20/KVIQ-10 are similar to those previously reported in healthy participants by Malouin et al. [25]. Similarly, the mean values for the MIQ-R were 22.29 (Visual), 21.71 (Kinesthetic), and 44.00 (Total), which are similar to those previously reported in healthy participants [37]. These observations confirm that the results for the KVIQ-20/KVIQ-10 and the MIQ-R gained in this study adequately reflect motor imagery abilities in individuals similar to assessments in previous reports.

We also investigated internal consistency with Cronbach’s alpha coefficients as an index of reliability. The results resemble assessments of internal consistency of the original KVIQ-20/KVIQ-10, which have been previously reported by Malouin et al. [25]. Therefore, our results indicate that the KVIQ-20/KVIQ-10 are highly reliable indices of motor imagery ability.

Additionally, we investigated criterion-related validity between the KVIQ and the MIQ-R as an index of validity. We observed significant positive correlations between the visual, kinesthetic, and total scores of the KVIQ-20 and the MIQ-R. Similar positive correlations were observed between the visual, kinesthetic, and total scores for the KVIQ-10 and the MIQ-R. The MIQ-R was developed by Hall & Martin [30] to assess motor imagery abilities in healthy individuals and athletes [38,39]. Previous research has indicated significant positive correlations between the KVIQ and MIQ-R subscales and total scores, similar to our results [36,40]. Therefore, our results indicate that the KVIQ is a highly valid index of motor imagery abilities.

Our study is limited in that we only investigated the KVIQ in healthy individuals. Therefore, the reliability and validity of the KVIQ among patients with disabilities remains unclear. However, previous studies have already reported the utility of the KVIQ for patients with stroke [22,29,35], Parkinson’s disease [29,36], multiple sclerosis [29,34], and brain tumors [29], as well as for elderly people [40,41]. Nonetheless, future research should investigate the usefulness of the Japanese version of the KVIQ for patients with disabilities. Moreover, there is no investigation of test-retest reliability of the KVIQ in this study. Therefore, further study is required to investigate test-retest reliability of the KVIQ in both healthy individuals and patients with disabilities. In addition, future study is required to investigate the neural mechanisms of KVIQ, and to examine brain activation in participants with good and poor motor imagery.

## Figures and Tables

**Table 1:** Score for the KVIQ-20/KVIQ-10 and the MIQ-R.

Variable		Mean	SD
KVIQ-20	Visual (50)	37.00	5.69
	Kinesthetic (50)	37.21	6.40
	Total (100)	74.21	11.02
KVIQ-10	Visual (25)	18.18	3.07
	Kinesthetic (25)	18.75	2.81
	Total (50)	36.93	5.29
MIQ-R	Visual (28)	22.29	3.32
	Kinesthetic (28)	21.71	4.10
	Total (56)	44.00	6.38

KVIQ-20 or 10, Kinesthetic and Visual Imagery Questionnaire—long (20 items) or short (10 items); MIQ-R, Movement Imagery Questionnaire-Revised. KVIQ-20 total scores range from 20 to 100 (visual and kinesthetic subscale scores each range from 10 to 50). KVIQ-10 total scores range from 10 to 50 (visual and kinesthetic subscale scores each range from 5 to 25). MIQ-R total scores range from 8 to 56 (visual and kinesthetic subscale scores each range from 4 to 28).

**Table 2:** Internal consistency of the KVIQ-20/KVIQ-10.

	KVIQ-20		KVIQ-10	
	Visual	Kinesthetic	Visual	Kinesthetic
Cronbach α	0.88	0.91	0.78	0.77
95% CI	0.80–0.94	0.85–0.95	0.64–0.89	0.62–0.88
SEM	1.08	1.21	0.58	0.53
MDC	2.99	3.35	1.61	1.47

KVIQ-20 or 10, Kinesthetic and Visual Imagery Questionnaire - long (20 items) or short (10 items); CI, Confidence interval; SEM, Standard error of measurement, MDC, Minimal detectable change.

**Table 3:** Spearman’s rank correlation coefficients between scores for the KVIQ-20/KVIQ-10 and the MIQ-R.

Variable	Correlation Coefficient	*p*-Value
	(r)	
KVIQ-20 Visual—MIQ-R Visual	0.64	<0.01
KVIQ-20 Kinesthetic—MIQ-R Kinesthetic	0.77	<0.01
KVIQ-20 Total—MIQ-R Total	0.86	<0.01
KVIQ-10 Visual—MIQ-R Visual	0.62	<0.01
KVIQ-10 Kinesthetic—MIQ-R Kinesthetic	0.78	<0.01
KVIQ-10 Total—MIQ-R Total	0.90	<0.01

KVIQ-20 or 10, Kinesthetic and Visual Imagery Questionnaire—long (20 items) or short (10 items); MIQ-R, Movement Imagery Questionnaire-Revised.
